# Timing of semen cryopreservation: before or after processing?

**DOI:** 10.61622/rbgo/2024rbgo36

**Published:** 2024-04-09

**Authors:** Ana Paula de Souza Kussler, Ivan Cunha Bustamante, Elisa Negri, Edison Capp, Helena von Eye Corleta

**Affiliations:** 1 Universidade Federal do Rio Grande do Sul Faculdade de Medicina Porto Alegre RS Brazil Faculdade de Medicina, Universidade Federal do Rio Grande do Sul, Porto Alegre, RS, Brazil.; 2 Generar Reprodução Humana Porto Alegre RS Brazil Generar Reprodução Humana, Porto Alegre, RS, Brazil.; 3 Universidade do Vale do Taquari Lajeado RS Brazil Universidade do Vale do Taquari, Lajeado, RS, Brazil.; 4 Hospital de Clínicas de Porto Alegre Porto Alegre RS Brazil Hospital de Clínicas de Porto Alegre, Porto Alegre, RS, Brazil.

**Keywords:** Semen, Sperm count, Spermatozoa, Centrifugation, Density gradient, Cryopreservation, Sperm wash, Seminal plasma

## Abstract

**Objective:**

Seminal cryopreservation causes significant damage to the sperm; therefore, different methods of cryopreservation have been studied. The aim of the study was to compare the effects of density gradient processing and washing/centrifugation with seminal plasma removal for cryopreservation in semen parameters.

**Methods:**

Seminal samples of 26 normozoospermic patients were divided into 3 parts: with seminal plasma; after washing/centrifugation; and after selection through density gradient. The samples were cryopreserved for at least two weeks. Motility, sperm count, morphology and viability were evaluated before cryopreservation and after thawing.

**Results:**

Density gradient processing selected motile and viable sperm with normal morphology in fresh samples (p<0.05). Cryopreservation negatively affected all sperm parameters regardless of the processing performed, and even if the sperm recovery was lower in the density gradient after the thawing, progressive motility, total motility, viability and morphology remained higher (p<0.05).

**Conclusion:**

Cryopreservation significantly compromises sperm parameters (motility, morphology, viability). In normozoospermic patients, the density gradients select better quality spermatozoa compared to other processing methods; this benefit was kept after thawing.

## Introduction

Sperm cryopreservation is the most efficient approach in the preservation of male fertility and has become one of the essential elements of assisted reproductive technology (ART).^(1)^ Significant improvements in the survival of patients with cancer, and other medical conditions have been achieved in the last few decades.^(2)^ Recognition and adequate patient counselling before gonadotoxic therapies are necessary, and semen cryopreservation should be offered to men without offspring. Similarly, social sperm banking and preservation for gender dysphoria prior to affirmation procedures are becoming more common. Cryopreservation is also mandatory in heterologous semen banks to provide semen for assisted reproduction programs in cases of sub-fertile semen, azoospermia or homoaffective couples.^(3,4)^

Undoubtedly, semen cryopreservation offers practical benefits to the assisted reproduction routine. However, cellular cryoinjuries play an important role in the process.^(5)^ This phenomenon happens due to the formation of intra- and extracellular ice crystals, the chemical toxicity of cryoprotectants, osmotic stress and cold shock. During cryopreservation, sperm cells go through dramatic changes in intra- and extracellular components.^(5,6)^ The chemical, physical and osmotic effects of this process may result in a loss of structural integrity and functional capacity of up 50% of spermatozoa. Semen samples with sub-fertile parameters are particularly susceptible to cryo-damage, possibly reducing the capacity of fertilization when compared to normal samples.^(7,8)^

Although the standard method of semen freezing involves the entire sample, with seminal plasma, studies have shown that the processing and selection of high-quality spermatozoa before freezing, with the removal of seminal plasma containing non-viable spermatozoa, leukocytes, bacteria and debris, improves sperm quality after thawing.^(9-12)^ The intrauterine insemination-ready ("IUI-ready") method, which uses density gradient in donor samples with normal sperm parameters and a cryoprotectant based on glycerol and sucrose, shows an improvement in sperm parameters after thawing without processing, with the possibility of "ready" insemination in the uterine cavity after thawing.^(13-15)^

However, some disadvantages in carrying out the pre-cryopreservation process have been demonstrated, especially those related to cellular damage caused by manipulation/centrifugation,^(16-19)^ the removal of seminal plasma, which protects cells against oxidative attack,^(20,21)^ the presence of polyunsaturated fatty acids (PUFAs), which increase plasma membrane fluidity, enhancing the resistance of lipoproteins that maintain the lipid composition of the plasma membrane at low temperatures, and heparin-binding proteins (HBPs) that prevent heat shock and peroxidation.^(22,23)^

The objective of this study was to compare conventional freezing with freezing after two methods of semen processing (washed/centrifugation and density gradient) in samples with normal sperm parameters. The main sperm parameters (motility, sperm count, morphology and viability) were compared.

## Methods

A cross-sectional prospective study was performed.

Patients counselled for infertility investigations from April 2018 to October 2018 at the *Generar Reprodução Humana*, Brazil, and volunteers were invited to participate in the study. Samples with total concentrations of sperm less than 30×10^6^/mL, leucocyte counts more than 1×10^6^/mL and volumes lower than 2.0 mL were excluded. Semen samples were collected by masturbation from 56 consecutive patients or volunteers. Of these, 30 were excluded: 3 oligozoospermic, 1 oligoasthenoteratozoospermic, 1 oligoasthenozoospermic, 2 teratozoospermic, 6 leucocytospermic, 6 hypospermic, 10 exhibited normal seminal parameters, but had a volume that was too low for our study and 1 was excluded after thawing due to the low recovery of spermatozoa in the fraction of the sample submitted to the density gradient.

This study followed the Guidelines and Norms Regulating Research Involving Human Subjects (Resolution 466/12 of the National Health Council) and was approved by the Research Ethics Committee of the Research and Post-Graduate Group of the *Hospital de Clínicas de Porto Alegre* (17-0314). All volunteers provided written informed consent.

Semen samples were collected by masturbation after an abstinence period of 3 to 7 days. Semen analyses were performed following 30 minutes of liquefaction at room temperature. The pH, volume, appearance, concentration, motility, viability (eosin) and morphology were analyzed according to the 2010 World Health Organization (WHO)^(24)^ parameters. Sperm concentration and motility were measured using a Makler Chamber, morphology was assessed following WHO guidelines and viability was assessed using eosin 0.5%, where live sperm appear white and dead sperm with disrupted membranes had taken in the eosin stain and appear red.

**Figure 1:**
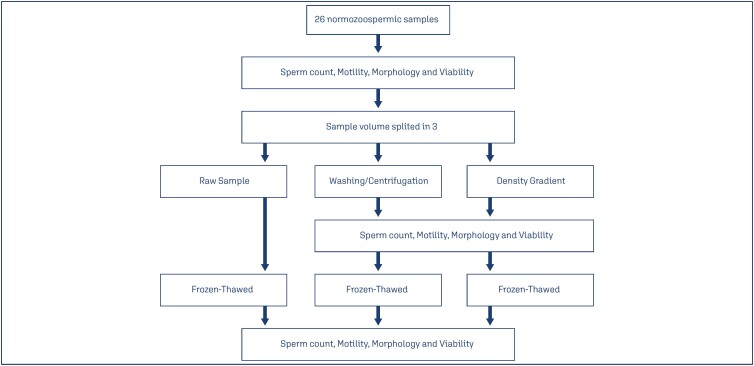
Study flowchart

Then, each sample was split into three parts: one to be cryopreserved with the seminal plasma, and the other two to be submitted to semen processing techniques, simple washing/centrifugation and density gradient. After processing, the two parts were analyzed again according to the WHO manual. At the end, an aliquot of each sample was cryopreserved (Figure 1).

In the washing/centrifugation group, the sample was diluted 1:1 with Modified HTF Medium-HEPES supplemented with 10% Serum Substitute Supplement (Irvine Scientific, Santa Clara, California) and centrifuged for 10 minutes at 300 g. The supernatant was removed, and the pellet re-suspended in an appropriate volume of the same medium (0.750 mL).

In the density gradient group, a density gradient was created by sequential pipetting of 1 mL of the 90% gradient and 1 mL of the 45% gradient (Irvine Scientific, Santa Clara, California), after which the sample was placed on the top of the gradient. The mixture was centrifuged for 20 min at 300 g and the viable sperm population was recovered from the 90% fraction and washed with Modified HTF Medium-HEPES supplemented with 10% Serum Substitute Supplement; the pellet was re-suspended in an appropriate volume of the same medium (0.750 mL).

First, the TEST-yolk Buffer cryoprotectant containing 12% glycerol (TYB, Irvine Scientific, Santa Clara, California) was thawed at room temperature, then the samples were added drop by drop in a 1:1 ratio and left there for ten minutes at room temperature for balancing. After this time, the samples were put into previously identified 0.250 mL straw (IMV) and sealed using the Poly Sealer P-200 (Fuji Impulse) sealer. The straws were then left horizontally in the liquid nitrogen vapor at a distance of 10 centimeters from the liquid surface for 10 minutes. Next, the same straws were directly immersed in liquid nitrogen. After at least 2 weeks, the straws were removed from the nitrogen tank and thawed on a heated plate at 37°C for 5 minutes. To remove the cryoprotectant, each fraction of the thawed specimen was subjected to washing/centrifugation separately and the same analyses were performed on the fresh samples.

To calculate the recovery rates the following formulas were used: Motile Spermatozoa Concentration: (Thawed motile spermatozoa concentration / Fresh sample motile spermatozoa concentration) × 100; Viable Spermatozoa: (Thawed viable spermatozoa percentage / Fresh sample viable spermatozoa percentage) × 100; Morphologically Normal Spermatozoa: (Thawed morphologically normal spermatozoa percentage / Fresh sample morphologically normal spermatozoa percentage) × 100.

The variables were described as the mean and standard deviation or standard error. In order to evaluate the effect of the freezing/thawing and processes on the parameters, the Generalized Estimates Equations (GEE) model was applied with the Bonferroni correction for multiple comparisons. For the variables with symmetric distribution, a linear model was used. For the discrete ones, the Poisson model (morphological changes: macrocephalous, short tail, bent tail and coiled) was used. The significance level adopted was 5% (p ≤ 0.05) and the analyses were performed in the SPSS program version 21.0.

## Results

### Patient characteristics

Samples from 26 patients/volunteers were included in this study. The mean age of the participants was 25.5 ± 6.36 years old, they had an average of 3.96 ± 1.03 days of sexual abstinence and the seminal volume of 3.85 ± 1.43 ml. According to the World Health Organization^(24)^ criteria, all patients presented sperm parameters within the normal range.

### Effect of sperm selection protocols and cryopreservation on seminal characteristics

Table 1 presents the effects of seminal preparations and cryopreservation on the samples. There was a decrease in the recovery of spermatozoa after processing, and the gradient presented the lowest concentration (37.73 ± 2.19 × 10^6^/mL; p<0.05). After thawing, the untreated (raw) and the washed/centrifugation sample groups showed a significant decrease in concentration, which did not occur with the gradient group (effect processing and effect cryopreservation: p<0.001 and effect processing x cryopreservation: p<0.164).

**Table 1:** Effect of sperm selection protocols and cryopreservation on seminal characteristics

Parameters		Raw	Washed	Density gradient
		Mean ± SEM	Mean ± SEM	Mean ± SEM
Sperm concentration (× 10^6^/ml)				
	Before freezing	55.61 ± 3.76^b^	48.08 ± 3.48^b^	37.73 ± 2.19^a^
	Frozen-thawed	46.28 ± 3.48^c^	41.98 ± 3.14^b^	31.63 ± 3.14^a^
	Difference (IC95%)	9.33 (2.35 a 16.32)	6.09 (1.15 a 11.03)	6.09 (-0.47 a 12.66)
	p-value	0.001	0.004	0.097
Progressive motility (a+b) (%)				
	Before freezing	46.42 ± 1.11^a^	45.07 ± 1.04^a^	60.69 ± 1.04^b^
	Frozen-thawed	22.11 ± 1.48^a^	22.69 ± 1.57^a^	31.30 ± 1.47^b^
	Difference (IC95%)	24.30 (19.57 a 29.94)	22.38 (19.97 a 27.79)	29.38 (24.12 a 34.64)
	p-value	<0.001	<0.001	<0.001
Total motility (a+b+c) (%)				
	Before freezing	60.46 ± 1.10^b^	56.26 ± 1.13^a^	73.50 ± 1.19^c^
	Frozen-thawed	32.76 ± 1.67^a^	32.53 ± 1.89^a^	41.26 ± 1.57^b^
	Difference (IC95%)	27.69 (23.16 a 32.22)	23.73 (18.36 a 29.10)	32.23 (26.79 a 37.66)
	p-value	<0.001	<0.001	<0.001
Non- progressive motility (%)				
	Before freezing	14.03 ± 0.85^b^	11.19 ± 0.80^a^	12.73 ± 0.87^a^,^b^
	Frozen-thawed	10.69 ± 0.47a	9.88 ± 0.56^a^	9.96 ± 0.55^a^
	Difference (IC95%)	3.34 (0.70 a 5.98)	1.30 (-1.38 a 3.99)	2.76 (0.007 a 5.53)
	p-value	0.003	1.0	0.049
Immotility (%)				
	Before freezing	39.57 ± 1.10^b^	43.76 ± 1.14^c^	26.50 ± 1.19^a^
	Frozen-thawed	67.19 ± 1.68^b^	67.30 ± 1.94^b^	58.80 ± 1.52^a^
	Difference (IC95%)	-27.61 (-23.03 a −32.19)	-23.53 (-18.06 a −29.01)	-32.30 (-27.00 a −37.61)
	p-value	<0.001	<0.001	<0.001

a,b,c Equivalent letters do not differ by the Bonferroni test at 5% significance

In the fresh samples, progressive motility was better after preparation by density gradient (60.69 ± 1.04%; p<0.05), which did not occur with the washing/centrifugation compared to the untreated samples (45.07 ± 1.04% and 46.42 ± 1.11%, respectively). Cryopreservation/thawing reduced both progressive and total motility in the three groups (frozen without treatment-raw: 22.11 ± 1.48 and 32.76 ± 1.67%; washing/centrifugation: 22.69 ± 1.57 and 32.53 ± 1.89% and gradient: 31.30 ± 1.47 and 41.26 ± 1.57%, respectively); the density gradient group had significantly better motility (effect processing, cryopreservation and processing x cryopreservation for both parameters: p<0.001). The percentage of immotile sperm decreased with density gradient selection (p<0.05), while washing/centrifugation resulted in an increase in these cells (p<0.05). After cryopreservation, there was a significant increase in immotile sperm in all groups; in the density gradient group, the percentage of immotile sperms was lower when compared to untreated and washed/centrifuged semen (p<0.05) (effect processing, cryopreservation and processing x cryopreservation: p<0.001). The analysis of progressive motile sperm concentration/mL in the samples showed a decrease in washing/centrifugation when compared to the untreated sample (p<0.05); there was no difference between the untreated and the gradient or washed/centrifugation and gradient groups. After cryopreservation, progressive motile sperm concentration decreased significantly in the 3 groups, with no difference between them (effect processing: p=0.001, effect cryopreservation: p<0.001 and effect processing x cryopreservation: p<0.013). The total motile sperm concentration/mL in the untreated sample was higher than in the fresh sample (p<0.05). Cryopreservation significantly decreased total motility (x10^6^/mL) in all 3 groups. In the unprocessed sample, the highest total motile sperm concentration was maintained in relation to the gradient group (p<0.05) and was similar to the washed/centrifugation group (p>0.05) (effects of processing, cryopreservation and processing x cryopreservation: p<0.001) (Table 2). The total and progressive motile sperm count (TMSC and PMSC) were higher in the fresh raw sample compared to the preparations. After thawing, the PMSC was not different between the three groups (p>0.05). The total motile sperm count (TMSC) after thawing of the untreated sample remained better compared to the gradient (p<0.05) (effect processing, cryopreservation and processing x cryopreservation for both parameters: p<0.001) (Table 2). As for sperm viability (Table 2), the gradient selected a higher number of viable spermatozoa when compared to the untreated group (77.50 ± 1.02 versus 68.38 ± 1.04%; p<0.05), while in the washed/centrifugation group there was a decrease compared to the untreated (62.00 ± 1.14%; p<0.05) samples. The cryopreservation decreased the viability of the cells in the three groups, with density gradient having the largest number of viable cells (44.23 ± 1.63% versus 39.61 ± 1.65% frozen without treatment and 38.00 ± 1.93% washed/centrifugation; effect of processing, cryopreservation and processing x cryopreservation: p<0.001).

**Table 2:** Effect of sperm selection protocols and cryopreservation on motile spermatozoa concentration (PMSC-TMSC), viability and normal morphology

Parameters	Raw	Washed	Density gradient
	Mean ± SEM	Mean ± SEM	Mean ± SEM
Progressive motile sperm concentration PMSC (× 10^6^/ml)			
Before freezing	25.71 ± 1.97_b_	21.62 ± 1.61**a**	22.95 ± 1.45_a.b_
Frozen-thawed	11.14 ± 1.39^a^	10.27 ± 1.21^a^	10.60 ± 1.36^a^
Difference (IC95%)	14.57 (9.97 a 19.16)	11.34 (8.25 a 14.44)	12.35 (8.70 a 15.99)
p-value	<0.001	<0.001	<0.001
Total motile sperm concentration TMSC (× 10^6^/ml)			
Before freezing	34.12 ± 2.67^b^	27.27 ± 2.18^a^	27.85 ± 1.78^a^
Frozen-thawed	16.19 ± 1.81^b^	14.71 ± 1.69^a.b^	13.85 ± 1.73^a^
Difference (IC95%)	17.92 (12.37 a 23.48)	12.56 (9.04 a 16.07)	13.99 (9.72 a 18.26)
p-value	<0.001	<0.001	<0.001
PMSC (× 10^6^/ml)			
Before freezing	95.72 ± 8.52^b^	16.44 ± 1.17^a^	17.21 ± 1.09^a^
Frozen-thawed	5.56 ± 0.69^a^	5.13 ± 0.60^a^	5.29 ± 0.68^a^
Difference (IC95%)	90.15 (65.71 a 114.58)	11.31 (8.83 a 13.78)	11.91 (9.38 a 14.44)
p-value	<0.001	<0.001	<0.001
TMSC (× 10^6^/ml)			
Before freezing	122.82 ± 12.21^b^	20.75 ± 1.60^a^	20.89 ± 1.33^a^
Frozen-thawed	8.09 ± 0.90^b^	7.35 ± 0.84^a.b^	6.94 ± 0.86^a^
Difference (IC95%)	114.73 (79.89 a 149.56)	13.40 (10.36 a 16.44)	13.94 (11.00 a 16.87)
p-value	<0.001	<0.001	<0.001
Live Sperm (%)			
Before freezing	68.38 ± 1.04^b^	62.00 ± 1.14^a^	77.50 ± 1.02^c^
Frozen-thawed	39.61 ± 1.65^a^	38.00 ± 1.93^a^	44.23 ± 1.63^b^
Difference (IC95%)	28.76 (24.68 a 32.85)	24.00 (19.44 a 28.55)	33.26 (28.76 a 37.77)
p-value	<0.001	<0.001	<0.001
Normal Morphology (%)			
Before freezing	14.88 ± 0.75^a^	14.30 ± 0.77^a^	20.50 ± 0.84^b^
Frozen-thawed	11.65 ± 0.44^a^	11.53 ± 0.48^a^	15.88 ± 0.61^b^
Difference (IC95%)	3.23 (1.23 a 5.22)	2.76 (0.72 a 4.81)	4.61 (1.87 a 7.35)
p-value	<0.001	0.001	<0.001

Recovery rates after sample freezing and thawing of the 3 groups are shown in table 3. No significant difference was found between the groups for progressive and total motile recovery or in the concentration of motile spermatozoa with progressive and total motility. The recovery rate of viable and morphologically normal spermatozoa was significantly higher in the samples after washing/centrifugation, with a higher recovery rate compared to samples after density gradient.

**Table 3:** Motility recovery, motile sperm concentration, viability and morphology rate

Parameters	Raw	Washed	Density gradient	Effects (p-value)
	Mean ± SEM	Mean ± SEM	Mean ± SEM	Processing
Progressive motility recovery rate (%)	47.87 ± 3.24^a^	50.85 ± 3.68^a^	52.08 ± 2.58^a^	0.060
Total motility recovery rate (%)	53.99 ± 2.55^a^	57.71 ± 3.07^a^	56.41 ± 2.26^a^	0.085
Progressive motile sperm concentration recovery rate (%)	43.26 ± 4.40^a^	45.51 ± 3.62^a^	44.71 ± 4.23^a^	0.659
Total motile sperm concentration recovery rate (%)	47.43 ± 4.05^a^	51.60 ± 3.48^a^	48.30 ± 4.35^a^	0.228
Viability recovery rate (%)	58.33 ± 2.05^a^.^b^	60.93 ± 2.78^b^	57.06 ± 1.98^a^	0.021
Morphology recovery rate (%)	80.63 ± 3.27^a^.^b^	82.74 ± 3.52^b^	79.26 ± 3.66^a^	0.020

a,b Equivalent letters do not differ by the Bonferroni test at 5% significance

The gradient kept more cells with normal forms (p<0.05; effect processing, cryopreservation and processing x cryopreservation: p<0.001) (Table 3). The main morphological changes between the processes and after cryopreservation are presented in table 4. Seminal processing caused morphological changes in spermatozoa, with an increase in macrocephalic heads in the group after density gradient and short tail and broken tail in both groups after processing (p<0.05). There was an increase in macrocephalic heads, broken and curled tails after freezing and thawing in the three groups (p<0.001).

**Table 4:** Morphological changes related to sperm processing and cryopreservation

Parameters		Raw	Washed	Density gradient
		Mean ± SEM	Mean ± SEM	Mean ± SEM
Macrocephalous				
	Before freezing	4.00 ± 0.56^a^	3.73 ± 0.55^a^	4.88 ± 0.69^b^
	Frozen-thawed	8.92 ± 1.09^a^	9.30 ± 1.09^a^	9.26 ± 1.06^a^
	Difference (IC95%)	-4.92 (-2.50 a −7.34)	-5.57 (-3.34 a −7.80)	-4.38 (-2.20 a −6.56)
	p-value	<0.001	<0.001	<0.001
Short Tail				
	Before freezing	1.76 ± 0.19^a^	3.73 ± 0.39^b^	3.92 ± 0.36^b^
	Frozen-thawed	3.65 ± 0.32^a^	3.69 ± 0.30^a^	3.69 ± 0.32^a^
	Difference (IC95%)	-1.88 (-0.88 a −2.88)	0.038 (-0.89 a 0.97)	0.23 (-0.64 a 1.11)
	p-value	<0.001	1.00	1.00
Bent tail				
	Before freezing	3.26 ± 0.30^a^	5.69 ± 0.36^c^	4.80 ± 0.29^b^
	Frozen-thawed	9.26 ± 0.49^a^	9.19 ± 0.44^a^	9.61 ± 0.45^a^
	Difference (IC95%)	-6.00 (-4.33 a −7.66)	-3.50 (-2.03 a −4.96)	-4.80 (-3.21 a −6.40)
	p-value	<0.001	<0.001	<0.001
Coiled tail				
	Before freezing	7.03 ± 0.83^a^.^b^	8.34 ± 0.92^b^	5.61 ± 1.02^a^
	Frozen-thawed	16.19 ± 1.18^a^	17.34 ± 1.42^a^	15.30 ± 1.32^a^
	Difference (IC95%)	-9.15 (-6.26 a −12.04)	-9.00 (-6.02 a −11.97)	-9.69 (-6.68 a −12.69)
	p-value	<0.001	<0.001	<0.001

a,b,c Equivalent letters do not differ by the Bonferroni test at 5% significance

## Discussion

Freezing semen is the standard method for male fertility preservation. Although advances have been achieved in this area, lethal and sub-lethal cryoinjury are associated with a 50% reduction in vitality and remain a major challenge.^(16,25)^ In this paper, the performance of different semen processing methods and sperm parameters in cryopreservation were evaluated.

Before any artificial reproduction technique (ART), a sperm selection technique such as density gradient or swim up should be performed. These techniques mimic some of the natural selection processes that occur in the female reproductive tract.^(26)^ The purpose is to improve seminal quality by selecting mobile, viable sperm with normal morphology and intact DNA in addition to removing seminal plasma, debris, immotile sperm, leukocytes, or immature germ cells and other substances deleterious for sperm viability.^(22, 26,27)^

The role of different preparations on DNA integrity remains controversial, some studies do not report impacts of preparations techniques^(28,29)^ while others report an increase in DNA damage^(30-32)^

The density gradient processing was effective in the selection of motile, viable and normal spermatozoa before freezing; this difference was kept after thawing process, as demonstrated in previous studies.^(9,12,13,33-35)^ Despite the lower concentration of spermatozoa after the gradient, which is a known effect of sperm selection techniques, the progressive motile sperm concentration and PMSC (progressive motile sperm count) were comparable between the treatments after the thawing process, indicating that the gradient method plays an important role for normozoospermic patients doing intrauterine insemination (IUI) and in storing in semen banks.^(14,15,29,36)^

The recovery rate (morphology and viability) after cryopreservation in the gradient samples was lower when compared to the washing/centrifugation group and were similar to traditional freezing (untreated samples frozen with seminal plasma), which is in contrast to the findings of Donnelly et al.,^(36)^ which showed lower recovery rates of morphology in the gradient over traditional freezing. The mechanical damage of the large manipulation, and the two centrifugations used in the gradient could be responsible for the worsening recovery rates.

Although previous studies described better recovery rates (motility and concentration) in the gradient group compared to the traditional method.^(14,32)^ or swim up,^(9)^ our results showed similar recovery rates for motility and progressive motile sperm concentration among all preparations, suggesting that the best recovery rate was due to the selection made by the gradient before freezing.

The protective effect of seminal plasma, its antioxidant capacity, and the action of polyunsaturated fatty acids (PUFAs) on the fluidity of the plasma membrane and to heparin-binding proteins (HBPs) that prevent heat shock and peroxidation was mentioned in some papers.^(22,23,37)^ Grizard et al.,^(16)^ in 1999 and by Fabozzi et al.,^(38)^ in 2016 studied the washing/centrifugation process before sample freezing, aiming to remove seminal plasma. They showed significantly lower semen parameters at thawing compared to freezing with seminal plasma and density gradient.^(34)^ Our results did not show a difference between the traditional and the washing/centrifugation methods in relation to motility, viability and morphology, among others.^(39-41)^ In our study, normozoospermic samples processed by the density gradient presented significantly higher seminal parameters. Cryopreservation affected the sperm parameters, but the impact of different processing methods was similar, raising the hypothesis that the cryoprotective role of seminal plasma is not essential in normozoospermic samples. Samples with better parameters prior to cryopreservation preserved their superiority after thawing. In the samples with normal semen parameters, the cryopreservation with previous density gradient selection proved to be effective and may be a safe option for homologous freezing or sperm freezing bank.
